# Economic evaluation of policy initiatives in the organisation and delivery of healthcare: a case study of gastroenterology endoscopy services

**DOI:** 10.1186/1478-7547-12-7

**Published:** 2014-03-05

**Authors:** David Cohen, M Fasihul Alam, Nishma Patel, Wai-Yee Cheung, John G Williams, Ian T Russell

**Affiliations:** 1 Faculty of Life Sciences and Education, University of South Wales, Pontypridd CF37 1DL, UK; 2 College of Medicine, Swansea University, Singleton Park, Swansea SA2 8PP, UK

**Keywords:** Cost effectiveness, Evaluation, Health policy, Modernisation, Endoscopy, Cost

## Abstract

**Background:**

Complex clinical interventions are increasingly subject to evaluation by randomised trial linked to economic evaluation. However evaluations of policy initiatives tend to eschew experimental designs in favour of interpretative perspectives which rarely allow the economic evaluation methods used in clinical trials. As evidence of the cost effectiveness of such initiatives is critical in informing policy, it is important to explore whether conventional economic evaluation methods apply to experimental evaluations of policy initiatives.

**Methods:**

We used mixed methods based on a quasi-experimental design to evaluate a policy initiative whose aim was to expedite the modernisation of gastroenterology endoscopy services in England. We compared 10 sites which had received funding and support to modernise their endoscopy services with 10 controls. We collected data from five waves of patients undergoing endoscopy. The economic component of the study compared sites by levels of investment in modernisation and patients’ use of health service resources, time off work and health related quality of life.

**Results:**

We found no statistically significant difference between intervention and control sites in investment in modernisation or any patient outcome including health.

**Conclusions:**

This study highlights difficulties in applying the rigour of a randomised trial and associated technique of economic evaluation to a policy initiative. It nevertheless demonstrates the feasibility of using this approach although further work is needed to demonstrate its generalisability in other applications. The present application shows that the small incentives offered to intervention sites did not enhance modernisation of gastroenterology endoscopy services or improve patient outcomes.

## Background

Clinical interventions are increasingly evaluated by randomised controlled trials (RCTs). The methods of economic evaluation which are used alongside RCTs are becoming well established e.g. [1,2]. In contrast evaluators of policy initiatives tend to eschew experimental designs, advocating more interpretative perspectives [3] and adopting context-dependent approaches like ‘realistic evaluation’ [4]. These are less amenable to the rigorous economic evaluation methods used in clinical trials.

In recent years, however, there have been efforts to extend the rigour of the RCT to the evaluation of clinical interventions that are more complex than single treatments. For example, the UK Medical Research Council (MRC) has developed a framework for the design and evaluation of ‘complex interventions’ which includes a definitive RCT [5]. Policy initiatives are arguably even more complex than complex clinical interventions. Given the need for evidence of the effectiveness of policy initiatives, however, the rigour of the RCT is still the goal. This paper describes a study which evaluated a policy initiative using a quasi-experimental approach and economic appraisal.

### The case study

In 2002, the National Health Service Modernisation Agency (NHSMA) initiated its Modernising Endoscopy Services (MES) programme to help gastroenterology departments in England modernise their endoscopy services. The specific purpose of the MES programme was to improve waiting times and throughput of endoscopy services by better matching demand and supply in endoscopy units and thus improve clinical outcomes. Following a pilot study, NHSMA chose 26 sites to receive £30,000 to fund approved service redesign plans and provided them with specially designed data software (Toolkit™) to analyse their endoscopy services, together with training and support from the MES itself. The Toolkit™ required the daily input of the number of referrals, patients waiting and lost appointment slots by procedure type and reason. These data were used by the endoscopy staff to monitor services and were uploaded to the MES programme for external analysis. Sites whose applications were unsuccessful were still eligible to use the Toolkit™ outside the MES programme.

This paper describes the methods and results of applying standard economic appraisal principles to evaluate the MES project. The final section discusses how the economic evaluation of this policy initiative differed from that in traditional clinical trials.

## Methods

The National Institute for Health Research funded the ENIGMA study (‘Evaluating New approaches In Gastroenterology initiated by the Modernisation Agency) through its Service Delivery and Organisation Programme to conduct an independent evaluation of the impact of the MES programme on services, patients and professionals [6]. ENIGMA adopted a quasi-experimental approach by selecting and recruiting random samples of ten of the 26 ‘intervention’ sites chosen by the NHSMA and ten of the 70 ‘control’ sites whose applications for funding to support modernisation had been unsuccessful; we stratified both samples by number of beds in the hospital.

We collected data from patients referred to these sites for endoscopy across five waves from April 2004 to April 2006. We assessed 9,154 patients for eligibility and invited 7,974 to participate; 3,818 consented to take part. ENIGMA gained ethical approval from the Wales Multi-Centre Research Ethics Committee and research governance approval from each site.

The controlled nature of ENIGMA made it a good candidate for economic evaluation. It addressed five economic questions:

1. What was the cost of modernisation in each site?

2. Did modernisation costs differ between intervention and control sites?

3. Was there a difference in use of other NHS resources between intervention site patients and control site patients?

4. Was there a difference in time off work between intervention and control site patients?

5. Was there a difference in health outcomes as measured by quality adjusted life-years (QALYs) between intervention and control site patients?

These are standard economic evaluation questions to address rigorously when undertaking economic evaluation alongside clinical trials. Attempting to apply them to a policy initiative, however, created numerous difficulties which often required creativity to overcome.

### Economic methods

The main economic analysis was a cost-utility study comparing total NHS costs with effects in terms of QALYs.

### Cost of modernisation

In economic evaluation alongside clinical trials, estimation of direct intervention costs, i.e. the value of the resources used in the provision of the intervention, is normally straightforward in well-defined interventions. Although the MES project sought to modernise endoscopy services, we could not find a widely agreed definition of ‘modernisation’, on which the cost of the intervention depends. As the stated challenge of the final phase of the MES project was to “introduce new ways of working”, for purposes of ENIGMA we defined modernisation as changes in working practices. Examples of such modernisation thus included changing methods (e.g. new IT systems to manage waiting lists), altering staff skill mix (e.g. training nurses to perform endoscopy), purchasing different equipment (e.g. long term decontamination units) and setting up new processes to monitor progress (e.g. modernisation team meetings). However we did not consider doing more of the same, for example by increasing staff numbers, as modernisation, although this clearly represents service *improvement*.

As many modernising changes began before the start of the evaluation we could not specify costs at the level of precision normally possible in prospective monitoring of resource use in economic evaluations alongside clinical trials. Instead, we estimated the resource costs of modernisation via two rounds of semi-structured interviews with key personnel at each study site, completed one year apart, early in 2006 and 2007. We asked sites to identify the individual with most knowledge of all aspects of the endoscopy service at that site to act as our informant. In response they nominated a range of managerial (e.g. Endoscopy Manager, Clinical Services Manager) and clinical (e.g. Consultant, Nurse Consultant, Endoscopy Sister) staff.

Before the first interview, we asked each site to provide relevant documentary sources describing its modernisation efforts and related resource consequences. We reviewed these and sent summaries to the selected informants before their first interview. These began with an explanation of principles to ensure that respondents had a clear understanding of what we considered a cost of modernisation. The second interview gave them the opportunity to respond to queries from the first interview and identify developments since the first visit.

Following standard methods of economic evaluation, we then measured resources which had been identified as contributing to modernisation in relevant natural or physical units and valued them using local data or national sources. In particular we valued staff hours according to grade; where we could not be sure of the grade, we estimated costs from the mid-point of the scale for similar advertised jobs [7].

Costs of off-site training courses undertaken by staff explicitly for modernisation included the money paid for courses where known, or else the cost for similar courses from web sources [8], plus the value of the trainee's time. In-house training included time of trainer and trainee, again valued according to grade. Where training led to staff re-grading, we included the extra cost of the re-graded post in the costs of modernisation.

We used the equipment costs incurred by sites, or else the most frequently incurred cost for similar equipment by other sites. We considered both training and equipment as one-off investments providing a flow of benefits over time and amortised all such costs by assuming five-year lifetimes and using a universal discount rate of 3.5% [9]. We also undertook a sensitivity analysis to test the effect of 10-year lifetimes on the costs of training and equipment. We adjusted all costs to common 2006 prices using the Health Service Price Index.

### Patients’ use of other health service resources

Modernising endoscopy services may also have indirect consequences for other health service resources. For example, shorter waiting times could affect patients’ use of General Practitioner (GP) services. We therefore collected data on patients’ use of other NHS services by questionnaires to patients recruited in five waves beginning in April 2004 and ending in April 2006. Each wave completed questionnaire at referral (Baseline Questionnaire – BQ), immediately after the procedure (Post Procedure Questionnaire – PPQ) and twelve months thereafter (12MQ). The time between being referred and undergoing the procedure varied between patients. We accepted returned questionnaires until the end of 2007. Where data were missing for the 12MQ, the last case carried forward method was adopted. For example where BQ (time point 0) and PPQ (time point 1) data were present but 12MQ (time point 2) data were missing, data were carried forward from the PPQ. A further weighting of the carried forward PPQ, based on the relative relationship of PPQ to 12MQ data, was not adopted as it was anticipated to be unlikely to have any major impact on the results.

When we undertook ENIGMA, there was little guidance how to collect resource use by patient recall, e.g. [10]. We therefore adapted a patient recall questionnaire which we had previously used successfully [11]. Questions asked patients about visits to GP surgeries and outpatient clinics, home visits by health professionals, hospital admissions including as a day case, and drugs prescribed – all within the three-month period before completing each questionnaire. Although use of a 3 month period produces gaps in the data, it has the advantage of providing more accurate patient recall of resource use than over longer periods [12]. Accuracy was considered more important than completeness as the focus here is on differences between groups. Table 1 shows the unit costs of these resources. Given the short timescale, we did not discount these costs.

**Table 1:** Unit costs and sources

**Data item**	**£ unit cost**	**Source**
GP (surgery visit)	£21	[13]
Nurse (surgery visit)	£8	[13]
GP (home visit)	£60	[13]
Nurse (home visit)	£11	[13]
Social services/Social worker	£60	[13]
Support worker	£11	[13]
Consultant	£73	[13]
Dietician/Nutritional nurse/Nutritional therapy session	£35	[14]
Physiotherapist session	£36	[14]
Paramedics/Ambulance	£161	[14]
Chiropodist/Podiatrist	£31	[14]
Occupational therapist	£36	[14]
Chiropodist/Podiatrist	£31	[14]
Colonoscopy	£352	[14]
Flexible sigmoidoscopy	£279	[14]
Gastroscopy	£275	[14]
Day case endoscopy	£457	[14]
Endoscopy slot	£2727	[14]*
Inpatient per day: medical	£269	[15] (inflated)
Outpatient episode	£96	[15] (inflated)
Avg. weekly earnings male	£105	[16]
Avg. weekly earnings female	£81.20	[16]
Drugs		Priced individually from [17]

*Slot = 12 points. Cost of slot based on assumed ½ session for colonoscopy (2 points) and ½ session for sigmoidoscopy/gastroscopy (1 point) per procedure.

### Value of patients’ lost productivity

Economic appraisals need to specify the perspective of their evaluations. In the UK, the National Institute for Health and Care Excellence (NICE) guides the cost-effective use of the National Health Service (NHS) budget by recommending which treatments the NHS should provide. It therefore prefers the perspective of NHS and personal social services [1].

Although there are also theoretical arguments to support this narrower perspective [18] we additionally assessed time off work by service uses as this might be reduced by improved service delivery. This was done by asking questions about time lost from work in BQ, PPQ and 12MQ. We compared lost work time and its value, estimated using average male and female earnings from Table 1.

### Quality of life and statistical analysis

For economic evaluation, our primary outcome was health-related quality of life measured by the EQ-5D [19], which are then converted into QALYs. We undertook analysis of covariance (ANCOVA) with QALY scores as dependent variable, group (intervention or control) as primary independent variable, and age, gender, procedure type, degree of urgency, waiting time (i.e. time between BQ and PPQ), NHS resource use before baseline, number of beds and teaching status of hospital, wave of recruitment and innovation scores as covariates.

Recruiting a large sample across many sites or ‘clusters’ carries a risk of losing statistical power through intra-site correlation. We therefore augmented analysis of covariance with multi-level modelling [20] to address this issue, in particular in costs at PPQ and 12MQ, using MLWin version 2.0 [21].

As PPQ and 12MQ questionnaires varied in the length of waiting time from baseline, an ANCOVA based adjustment was carried out on data from these two questionnaires to account for baseline effects across all resource use items as well as for costs. To address the inevitable skewness in cost data, we also used non-parametric bootstrapping [22] when analysing costs.

## Results

### Cost of modernisation

Table 2 shows the estimated mean costs of modernisation. As it proved difficult to specify exactly when many modernisation activities began, we treated all training and equipment costs that occurred during the 12-month modernisation period between site visits as ‘initial’ costs occurring simultaneously. We adopted the same approach to ‘one-off’ costs of other activities which finished when modernisation was complete.

**Table 2:** Mean (SD) costs, activity and marginal costs

	**Equipment**	**Training**	EAC*	**One off costs**	**Recurring**	**Initial cost**	**2004 activity**	**Initial marginal cost/patient**	**Subsequent year cost**	**2005 activity**	**Subsequent year marginal cost/patient**
**Intervention (I)**	£33432 (66674)	£7727 (5747)	£9116 (14987)	£51888 (74323)	£69949 (68537)	£130,954 (£81,714)	5392 (1524)	£26.62 (16.71)	£79, 066 (£71,777)	5244 (1138)	£17.43 (16.40)
**Control (C)**	£72346 (100821)	£6146 (9656)	£17385 (21949)	£40763 (37321)	£75825 (83335)	£133,972 (£100,928)	4417 (1703)	£33.88 (24.23)	£93,209 (£95,295)	4787 (1848)	£20.91 (19.04)
**Difference C–I (p value, 95% CI)**						£3,019 (.07,-£88,745 to £94,783)		£7.26 (.74, -£13.55 to £28.05)	£14,143 (.74,-£70,160 to £98,447)		£3.48 (.42,-£14.27 to £21.24)

*Equivalent annual cost.

P values (95% CI) for totals.

Table 2 shows total initial costs in column 7. This is the sum of the Equivalent Annual Cost (EAC) of equipment and training (column 4), the one-off costs (column 5) and the annual recurring costs such as permanent new modernisation-related posts (column 6). Dividing these initial costs by the activity (i.e. average number of endoscopies) in 2004 yields the marginal initial cost per patient i.e. the additional cost per patient due to modernisation. Table 2 also shows costs in subsequent years, namely recurring costs plus EAC of training and equipment, the activity in 2005 and the resulting marginal subsequent cost per patient-year.

All costs varied widely: equipment from zero to £260,000; training from £400 to £31,000; and in one-off costs from zero to £245,000. As an example of the latter, one site produced a ‘modernisation initiative endoscopy list’ and cleared it by sending patients to the local private hospital (£68,000).

The mean total initial cost was £131,000 (SD = £82,000) for intervention sites and £134,000 (SD = £101,000) for control sites. The difference of £3,000 in favour of the intervention was not statistically significant (95% CI from -£88,745 to £94,783). The mean total cost for subsequent years was £79,000 (SD = £72,000) in intervention sites and £93,000 (SD = £95,000) in control sites. The difference of £14,000 in favour of the intervention was also not significant (95% CI from -£71,000 to £98,000).

There were also no statistically significant differences in marginal costs per patient between intervention and control sites either in initial or in subsequent years. The mean difference in initial marginal costs was £7 in favour of the intervention (95% CI from -£14 to £28) and the difference in marginal costs for subsequent years was £3 in favour of the invention (95% CI from -£14 to £21). The sensitivity analysis assuming a 10-year lifetime for training and equipment did not change the relative costs of intervention and control sites and the differences remained not statistically significant.

### Patients’ use of other health service resources

After the first wave of patient recruitment, one intervention and one control site were withdrawn from the study because they were unable to comply with the strict patient recruitment criteria [6]. Patient-level data on use of NHS resources, time off work and quality of life were thus available for 18 of the 20 recruited sites (9 intervention, 9 control). A total of 3,818 BQs, 2,940 PPQs and 2,588 12MQs were available for complete case analyses (CCA). Imputation of missing data increased the number of analysable PPQs to 3055 and 12MQs to 3039. However, comparison of mean differences between patients in intervention and control sites showed little difference in results between methods. For example, all differences that were statistically significant (*p* <0.05) in the CCA remained significant with imputation and no non-significant results became significant. Thus although the imputation used the full dataset, it had little effect on results. It was therefore decided to report results only from the CCA.

Results of the multi-level modelling (MLM) did not show any statistically significant effects between sites on the five selected cost variables in either PPQ or 12MQ. Estimates of intra-site correlation, which reflect the degree of clustering at site-level, did not show any statistically significant site effects for the observed resource use data. Accordingly we conducted no further MLM analyses and report the remaining results without the minimal adjustments for clustering.

Table 3 shows the raw costs of other NHS resources, split by wave and group. Table 4 shows mean differences in NHS costs, where baseline costs are bootstrapped and post procedure and end of follow up costs are adjusted for baseline differences and waiting time. Most differences were negative indicating that patients from intervention sites incurred lower costs but few differences reached statistical significance. All differences which were statistically significant at PPQ became non-significant at 12MQ. The only statistically significant difference at 12MQ was for drugs in Wave 4 (*p* = 0.04) but this was not seen in Wave 5.

**Table 3:** Mean cost (standard error) of other NHS resources

**Total primary care cost (£)**									
**Wave**	**Baseline (n = 3818)**			**Post procedure (n = 2940)**			**End of follow up (n = 2588)**		
	**Intervention mean (SE)**	**Control mean (SE)**	**P value**	**Intervention mean (SE)**	**Control mean (SE)**	**P value**	**Intervention mean (SE)**	**Control mean (SE)**	**P value**
W1	£68.59 (4.28)	£75.37 (5.03)	0.31	£61.81 (4.75)	£60.51 (3.80)	0.83	£45.51 (3.89)	£52.03 (4.03)	0.25
W2	£79.71 (6.67)	£74.32 (4.11)	0.49	£70.70 (5.76)	£68.61 (4.41)	0.77	£56.18 (5.69)	£57.00 (5.63)	0.92
W3	£72.48 (4.96)	£69.25 (3.72)	0.6	£50.41 (3.64)	£72.41 (5.12)	**0.00**	£43.07 (4.75)	£52.43 (7.71)	0.32
W4	£65.02 (3.83)	£84.13 (8.74)	**0.04**	£52.30 (3.25)	£63.67 (5.22)	0.06	£49.87 (4.51)	£52.84 (5.44)	0.67
W5	£69.95 (4.58)	£77.19 (6.17)	0.34	£53.15 (3.72)	£59.53 (3.79)	0.23	£37.15 (3.16)	£71.46 (17.77)	0.06
**Total secondary care cost (£)**									
W1	£262.78 (24.20)	£311.05 (25.17)	0.17	£691.44 (27.25)	£714.36 (31.44)	0.59	£227.74 (30.31)	£181.84 (21.34)	0.21
W2	£292.48 (25.91)	£336.44 (34.21)	0.31	£718.58 (34.03)	£694.83 (33.67)	0.62	£187.67 (24.52)	£257.31 (44.29)	0.18
W3	£263.71 (24.26)	£300.95 (31.32)	0.36	£626.22 (32.71)	£717.11 (30.52)	**0.04**	£151.09 (22.15)	£222.11 (31.16)	0.07
W4	£257.34 (20.92)	£301.73 (24.41)	0.17	£681.62 (29.51)	£698.94 (34.42)	0.70	£174.83 (27.07)	£265.52 (56.68)	0.15
W5	£302.09 (27.55)	£275.88 (23.89)	0.48	£653.91 (25.11)	£625.13 (25.92)	0.43	£162.94 (23.92)	£207.48 (30.28)	0.25
**Total cost of medication (£)**									
W1	£33.36 (3.13)	£35.81 (3.82)	0.62	£33.29 (2.99)	£44.80 (4.98)	**0.05**	£31.12 (3.32)	£43.05 (4.82)	**0.05**
W2	£27.03 (2.55)	£43.82 (4.01)	**0.00**	£29.07 (3.01)	£40.00 (3.28)	**0.02**	£30.89 (3.25)	£42.18 (5.57)	**0.02**
W3	£33.31 (3.15)	£40.17 (3.00)	0.12	£33.60 (3.03)	£42.92 (3.78)	0.06	£33.02 (3.55)	£45.87 (5.00)	**0.04**
W4	£36.36 (3.45)	£38.40 (3.13)	0.66	£36.23 (3.54)	£42.05 (4.33)	0.29	£36.25 (3.81)	£48.37 (4.70)	**0.05**
W5	£40.09 (3.78)	£42.32 (3.43)	0.66	£40.62 (4.26)	£41.66 (3.67)	0.86	£39.29 (5.73)	£46.65 (5.67)	0.36
**Total NHS cost**									
W1	£364.72 (25.37)	£422.24 (26.77)	0.13	£786.55 (29.11)	£819.66 (33.98)	0.46	£304.37 (31.42)	£276.92 (24.03)	0.48
W2	£399.22 (27.96)	£454.58 (35.44)	0.22	£818.35 (35.81)	£806.05 (35.42)	0.81	£274.74 (26.13)	£356.49 (47.97)	0.14
W3	£369.52 (26.58)	£410.38 (32.28)	0.34	£715.01 (34.55)	£835.02 (33.17)	**0.01**	£227.17 (24.09)	£320.42 (34.45)	**0.03**
W4	£358.72 (22.04)	£424.26 (27.07)	0.07	£770.15 (30.58)	£807.54 (36.32)	0.43	£260.95 (30.26)	£366.72 (58.19)	0.10
W5	£412.12 (28.86)	£395.39 (25.78)	0.67	£747.68 (26.67)	£726.31 (27.70)	0.58	£239.38 (26.24)	£325.59 (40.24)	0.07

BOLD: p ≤ .05.

**Table 4:** Comparison of bootstrapped mean difference (MD) in baseline and adjusted mean difference in post procedure and end of follow up costs by wave: intervention v control

**Wave**	**Baseline (n = 3818)**			**Post procedure (n = 2940)**			**End of follow up (n = 2588)**		
	**Bootstrapped MD (I – C)**	***p***	**95% CI**	**Adjusted MD (I – C)**	***p***	**95% CI**	**Adjusted MD (I – C)**	***p***	**95% CI**
**Primary care cost (£)**									
W1	-£6.74	0.15	-19.80 to 6.00	£5.15	0.33	-5.28 to 15.58	-£5.97	0.28	-16.72 to 4.79
W2	£5.35	0.75	-7.19 to 24.40	-£2.47	0.69	-15.92 to 9.99	-£1.28	0.87	-16.80 to 14.20
W3	£3.34	0.71	-7.90 to 16.11	-£21.6	**0.00**	**-32.50 to -10.80**	-£8.88	0.34	-27.10 to 9.31
W4	-£19.30	**0.02**	**-43.80 to -4.22**	-£9.16	0.11	-20.23 to 1.92	£0.87	0.90	-12.50 to 14.30
W5	-£7.28	0.17	-25.20 to 5.90	-£1.42	0.76	-10.65 to 7.82	-£35.6	0.06	-71.90 to 0.08
**Secondary care cost (£)**									
W1	-£48.30	0.08	-111.30 to 23.80	-£12.50	0.76	-91.70 to 66.60	£37.60	0.29	-33.40 to 108.60
W2	-£43.40	0.16	-122.50 to 48.40	£23.00	0.62	-66.70 to 112.70	-£62.60	0.19	-156.20 to 30.90
W3	-£37.60	0.17	-116.80 to 37.40	-£66.60	0.10	-146.20 to 12.09	-£62.90	0.10	-138.60 to 12.60
W4	-£44.30	0.08	-107.10 to 17.60	-£7.98	0.85	-89.10 to 73.20	-£58.60	0.33	-176.00 to 58.90
W5	£26.20	0.76	-45.80 to 98.70	£32.70	0.34	-34.90 to 100.40	-£48.60	0.22	-125.90 to 28.60
**Drug cost (£)**									
W1	-£2.46	0.31	-12.30 to 6.90	-£9.99	0.08	-21.01 to 1.10	-£10.60	0.06	-21.80 to 0.54
W2	-£16.90	0.06	**-26.50 to -8.20**	-£3.90	0.32	-11.70 to 3.80	-£5.90	0.20	-15.00 to 3.10
W3	-£6.90	0.33	-15.40 to 1.40	-£6.10	0.13	-14.10 to 1.80	£8.50	0.15	-19.90 to 2.90
W4	-£2.05	0.33	-10.50 to 7.60	-£5.00	0.24	-13.40 to 3.30	-£10.80	**0.04**	**-2110 to -0.50**
W5	-£2.23		-11.80 to 8.10	-£2.50	0.54	-10.0 to 5.50	-£6.40	0.40	-21.40 to 8.50
**Total NHS cost (£)**									
W1	-£57.50	0.06	-128.20 to 19.80	-£17.20	0.69	-101.60 to 67.30	£20.60	0.59	-54.70 to 95.80
W2	-£54.90	0.12	-137.30 to 5.90	£15.50	0.74	-77.00 to 108.00	-£66.00	0.19	-166.60 to 34.60
W3	-£41.10	0.16	-123.90 to 37.90	-£86.90	**0.04**	**-169.80 to -4.10**	-£80.10	0.06	-162.50 to 0.30
W4	-£65.60	**0.03**	**-135.08 to -3.10**	-£21.20	0.62	-105.60 to 63.30	-£64.00	0.30	-186.30 to 8.30
W5	£16.70	0.66	62.10 to 93.70	£27.20	0.45	-43.80 to 98.00	-£91.40	0.06	-187.30 to 4.50

BOLD: p ≤ .05.

### Value of patients’ lost productivity

Table 5 shows mean number of days off work and value of lost productivity by wave. There was a statistically significant difference in time off work in favour of the intervention in wave 4 (adjusted mean difference = -1.78 days; 95% CI from -3.47 to -0.09 days) but not in any other wave. When valued using relevant average earnings (male or female), however, the adjusted mean difference of -£27 was not statistically significant (95% CI from -£57 to £3). This result mirrors those seen for other NHS resources.

**Table 5:** Time off work and value of lost productivity by wave: intervention v control

	**Baseline (n = 3818)**			**Post procedure (n = 2940)**			**12 month post procedure (n = 2588)**		
	**M.D. (I – C)**	***p***	**95% CI**	**Adj. M.D. (I - C)**	***p***	**95% CI**	**Adj. M.D. (I - C)**	***p***	**95% CI**
**(D7)** Time off from work									
W1	-0.03	0.97	-1.55 to 1.49	0.27	0.61	-0.75 to 1.28	-0.48	0.57	-2.12 to 1.16
W2	-1.20	0.22	-3.11 to 0.71	0.49	0.56	-1.16 to 2.13	0.11	0.80	-0.70 to 0.91
W3	0.55	0.37	-0.64 to 1.74	-0.55	0.22	-141 to 0.32	-0.05	0.89	-0.85 to 0.75
W4	1.59	0.07	-0.13 to 3.32	-0.04	0.95	-1.26 to 1.18	-1.78	**0.04**	**-3.47 to -0.09**
W5	-0.82	0.28	-2.33 to 0.68	-1.04	0.31	-3.04 to 0.97	-0.16	0.82	-1.58 to 1.26
Cost of Time off from work									
W1	-£0.86	0.95	-27.50 to 29.20	£5.47	0.59	-14.40 to 25.30	-£9.80	0.57	-43.40 to 23.90
W2	-18.60	0.28	-52.60 to 15.30	£14.05	0.37	-16.90 to 45.00	£2.40	0.73	-11.60 to 16.50
W3	£12.30	0.29	-10.70-to 35.30	-£10.70	0.15	-25.40 to– 3.90	-£1.45	0.83	-14.80 to 11.90
W4	£32.00	0.05	-0.30 to 64.30	£1.60	0.88	-20.60 to 23.90	-£27.00	0.07	-56.70 to 2.60
W5	-£17.30	0.22	-44.90 to 10.20	-£20.70	0.29	-59.70 to 18.30	-£6.20	0.65	-33.20 to 20.70

BOLD: p ≤ .05.

Taken together, the results suggest that total costs may have been lower in intervention sites, but the difference was not statistically significant.

### Quality of life

Table 6 shows two statistically significant adjusted mean differences in QALYs: against the intervention in Wave 2 at 12 months; and favouring the intervention in Wave 5 immediately after the procedure. The differences in the other 8 analyses were not significant with five favouring the intervention group and three favouring the control group. The other quality of life measures used in ENIGMA replicated this finding that the MES initiative did not have a statistically significant impact on quality of life [6].

**Table 6:** Adjusted mean difference in QALY scores: PPQ (*) and 12MQ (**)

	**PPQ**		**12mPPQ**	
	**Adjusted mean difference (I – C)**	**P value (95% CI)**	**Adjusted mean difference (I – C)**	**P value (95% CI)**
W1	-0.006	0.48 (-.0210 to .010)	-0.007	0.15 (-.017 to .003)
W2	0.003	0.74 (-.014 to .020)	-0.012	**0.04** (-.023 to -.001)
W3	0.015	0.08 (-.002 to .031)	0.010	0.08 (-.001 to .021)
W4	0.003	0.75 (-.014 to .019)	0.001	0.92 (-.001 to .011)
W5	0.017	**0.04** (.001 to .032)	0.004	0.40 (-.006 to .015)

(*) = post procedure questionnaire.

(**) = 12 month post procedure questionnaire.

BOLD: p ≤ .05.

## Discussion

The economic questions addressed here were part of a larger study which examined the impact of the Modernising Endoscopy Services (MES) programme on a range of criteria by a variety of methods. These included: questionnaires to study sites about process data and innovation histories; questionnaires to patients about outcomes and waiting times; questionnaires to general practitioners with patients in ENIGMA about the modernisation of endoscopy services at their local study site; interviews to obtain the views of patients and professionals working at study sites; and focus groups to obtain the views of professionals working at non-study sites. Results from all methods were essentially consistent with no conflicting messages [6].

The quasi-experimental design of ENIGMA sought to evaluate whether a policy initiative based on a small financial contribution plus non-financial support from MES programme staff enhanced the modernisation of endoscopy services. We have previously succeeded in randomising Primary Care Trusts to evaluate a policy initiative to facilitate collaboration between general medical practices and community pharmacies in North and East Yorkshire [23,24]. Not surprisingly, however, the NHSMA preferred to choose 26 of the 80 applicant sites which they judged most likely to succeed. Hence the closest we could come to a rigorous quasi-experiment was by drawing stratified random samples from these 26 successful sites and the remaining 70 unsuccessful sites. If the choices made by NHSMA had been better than random, our evaluation would have been biased.

We also had to compromise when applying standard economic evaluation methods alongside our quasi-experiment. While the intervention took the clear form of support from MES, its aim was to expedite ‘modernisation’, for which there was no clear definition. Identifying which investments to include as costs of modernisation thus required an operational definition. So we chose to define modernisation as doing things *differently* rather than doing more of the same. We then relied on key personnel to decide whether each activity fitted that definition. Interviewees often had difficulty in accepting that this definition excluded some initiatives of which they were proud, for example creating new consultant and nursing posts which had reduced waiting times. However not all examples, were as clear; thus the need to rely on informants’ judgment whether an initiative represented modernisation or improvement was unavoidable and open to bias. It also proved difficult to isolate investments supported by the £30,000 provided to intervention sites from the other investments in modernisation.

Timing also posed several problems. Clinical trials normally introduce interventions at a defined time, whereas many study sites had started modernising long before MES began. This made it difficult to judge whether the cost of each modernisation activity fell within the relevant timeframe. Thus the wide variation in the equipment costs associated with modernisation, from zero to £260,000, depended on the situation at the start of the modernisation period. For example, one endoscopy service which reported no new equipment costs had recently occupied a new unit at a cost of some £2.5 million. Nevertheless, since its new equipment was in place before MES began, we could not attribute the costs of this equipment to MES. At the other extreme, one financially constrained service made no investments in new equipment. Similarly, the wide variation in training costs, from £444 to £31,100, reflected training needs at the start of the modernisation period.

The fact that the Department of Health launched MES at a time when modernisation was one of their key policy objectives for the NHS as a whole exacerbated these problems. Thus, despite our use of control sites to adjust for extraneous factors, the momentum of modernisation meant that the specific effects of an endoscopy-specific initiative were sure to be small. In addition, the policy context did not allow the study design to minimise contamination between groups as could have been done within an RCT. Control sites were not barred from using the Toolkit™ and while we do not know how many did so, any such use would dilute the intervention effect.

As a major aim of the study was to investigate whether standard methods of economic evaluation could be applied to a policy initiative, we intentionally applied a rigorous costing methodology. While the difficulties discussed above reduced the precision of our cost estimates, ENIGMA showed that many standard methods of economic evaluation could be applied without difficulty – in particular to the estimation of the cost of patients’ use of other health services and the value of patient level lost productivity and health related quality of life.

## Conclusions

This case study has shown that even for an initiative as unspecified as Modernising Endoscopy Services, it is possible to design an economic evaluation alongside a quasi-experiment, albeit with caveats about the conclusions drawn. This design enabled us to compare the effects of the MES programme with those of a plausible alternative.

Subject to those caveats, ENIGMA has concluded that small incentives such as those offered to intervention sites by the NHSMA do not stimulate further investment. Furthermore they do not affect patient outcomes, other demands on the NHS or losses to industry from sickness absence. While less rigorous designs can also draw such economic conclusions, they suffer from the weaknesses inherent in studies which do not define a comparator.

Our use of a case study to explore the broad issue of whether standard economic evaluation techniques can be applied to policy initiatives using quasi-experimental designs inevitably raises questions about the extent to which the messages are generalisable and further applications are clearly required.

## Competing interests

The authors declare that they have no competing interests.

## Authors’ contributions

DC, WYC, JGW and IR were involved in designing the study. DC drafted the manuscript. MFA carried out the main analysis and contributed to drafting the manuscript. NP was involved with literature search and finding costs figures. All authors approved the final manuscript.
